# Phylogenetic analysis of mammalian SIP30 sequences indicating accelerated adaptation of functional domain in primates

**DOI:** 10.1016/j.bbrep.2023.101631

**Published:** 2024-01-01

**Authors:** Ning Guo, Jeremy Raincrow, Chi-hua Chiu, Lei Yu

**Affiliations:** a Department of Genetics, Rutgers University, Piscataway, NJ, 08854, USA; b School of Biomedical Sciences, and Department of Biological Sciences, Kent State University, Kent, Ohio, 44242, USA; c Center of Alcohol & Substance Use Studies, Rutgers University, Piscataway, NJ, 08854, USA

**Keywords:** SIP30, Coiled-coil domain, Mammal gene, Neuropathic pain

## Abstract

SIP30, characterized by a coiled-coil functional domain, plays a key role in regulating synaptic vesicle exocytosis and is implicated in neuropathic pain resulting from peripheral nerve injury. Because neuropathic pain is studied in primates (including human), domesticated animals, and rodents, we conducted a phylogenetic analysis of SIP30 in selected species of these three groups of mammals. SIP30 exhibits a high degree of sequence divergence in comparison to its protein binding partners SNAP25 and ZW10, which show broad sequence conservation. Notably, we observed an increased rate of change in the highly conserved coiled-coil domain in the SIP30 protein, specifically within primates. This observation suggests an accelerated adaptation of this functional domain in primate species.

## Highlights

• SIP30 plays a pivotal role in regulating synaptic vesicle exocytosis and neuropathic pain.
• SIP30 exhibits greater sequence divergence compared to its binding partners.
• The coiled-coil domain shows an elevated rate of change only within primates, and not in domesticated animals or rodents.
• This suggests an accelerated adaptation of this functional domain in primates.

## 1 Introduction

SIP30 was initially identified as an interacting protein with ZW10 (Zeste White 10, a kinetochore protein) [1]. Subsequently, SIP30 was discovered to associate with SNAP25 and Rab3 proteins [2,3]. SNAP25 is a member of the SNARE family proteins crucial for vesicle exocytosis [4,5], and Rab3 proteins are important regulators of the same cellular process [6,7]. Like other SNARE proteins and SNARE-interacting proteins, SIP30 (also known as Zwint) contains a putative coiled-coil domain, important for its interaction with both SNAP25 and Rab3 [3].

The biological function of SIP30 has been associated with neuropathic pain [[8], [9], [10]]. Neuropathic pain, arising from damage or dysfunction of the nervous system [11], is characterized as a chronic, sustained painful condition [12,13]. Neuropathic pain tends to persist long after the primary injury has healed, and current treatment options remain inadequate for most patients [12,13]. In an effort to study the molecular changes in the nervous system underlying the manifestation of neuropathic pain, we identified SIP30 as an over-expressed gene in the lumbar spinal cord of the rodent neuropathic pain model of CCI (chronic constriction injury). We have also demonstrated a causal relationship between the over-expression of SIP30 and the manifestation of neuropathic pain behavior [[8], [9], [10]], and SIP30's role in synaptic vesicle exocytosis [14].

The patterns of SIP30 mRNA and protein expression corroborate with SIP30 involvement in neuropathic pain. SIP30 was expressed in the dorsal horn laminae of the spinal cord, where peripheral nociceptive inputs first synapse [8]. In animals experiencing neuropathic pain, SIP30 expression at both mRNA and protein levels increased in the lumbar enlargement section of the spinal cord. Notably, this increased SIP30 expression was observed only on the ipsilateral side of the spinal cord, indicating specificity related to neuropathic pain [8]. Additionally, SIP30 expression showed an increase in the rostral anterior cingulate cortex (rACC), and SIP30 inhibition in rACC led to the reversal of neuropathic pain-evoked aversion [10].

Neuropathic pain, a pathological condition of the nervous system, has been documented in rodents, domesticated animals, and human, and is commonly studied in both rodent models and human subjects [[15], [16], [17]]. In this study, we investigated SIP30 messenger RNA sequences in selected species of primates (including human), domesticated animals, and rodents. We compared SIP30 protein coding regions (CDS) as well as amino acid sequences. Our findings reveal a high degree of sequence divergence in SIP30 compared to its protein binding partners. Moreover, we observed an accelerated adaptation in the coiled-coil domain, the functional domain in SIP30, specifically in primates including humans.

## 2 Materials and methods

### 2.1 Sequence retrieval

Rat SIP30 cDNA sequence was used to search NCBI database with the Blastn algorithm. Sequences that showed significant similarities to the query sequence were categorized by species, and deduced protein coding regions were extracted. There are possible alternative splice variants of SIP30, which, in most of the cases, result in changes in the 5′ and 3’ non-translated region. When alternative splicing does cause changes in the open reading frame, the splicing variant that most resembles SIP30 sequences from other species, in terms of the number and the length of the exons, was used.

### 2.2 Phylogenetic tree construction

Gene trees were made using both nucleotide and deduced amino acid sequence alignments.

Model Selection: ModelTest v3 [18] was used to select the likelihood model for the nucleotide sequence data. The Bayesian information criterion (BIC) [19,20] was used to select the HKY + G model [21] with the following parameters: Base frequencies; A = 0.2844, C = 0.2519, G = 0.2947, T = 0.1690. Substitution model Ti/Tv ratio = 2.2233. Among site rate variation, proportion of invariable sites = 0; Gamma distribution shape parameter = 0.9665. MrModelTest v2 [22] was used to select the likelihood model for the nucleotide sequence data that could be implemented by MrBayes. The HKY + G model was used which, when implemented in MrBayes, gave a prior state frequency of dirichlet (1,1,1,1) and used the basic model nst = 2 with among site rate variation set to estimate rates based on a gamma shaped distribution.

Neighbor joining and maximum parsimony trees for both nucleotide and amino acid sequence data were constructed using PAUP* v4 [23]. Node confidence was assessed using 2000 bootstrap replications. A consensus tree was created using majority rule with a cutoff at 50 %. Maximum likelihood trees for the nucleotide sequence data were constructed using GARLI v0.951 [24]. The starting tree was obtained using heuristic search under the likelihood optimality criterion in PAUP* v4 [23] with settings as specified by ModelTest [18].

Bayesian trees for the nucleotide sequence data were constructed using MrBayes v3 [25] and the parallel version of MrBayes v3 [26] under the settings specified by MrModelTest (see Model Selection). Two independent Markov Chain Monte Carlo analyses were conducted with the following settings: number of generations was set to 1,000,000, sample frequency was taken every 1000 steps, number of chains was set to 4 and the temperature was set to 0.2. ‘Burnin’ was assessed after the run using the sum parameters command. The ‘burnin’ for the nucleotide analysis was set to 1, which is equal to the first 1000 steps of tree topologies. A majority rule consensus tree was created disregarding the ‘burnin’ trees using the sum trees command with a cut off of 0.50 posterior probability.

Bayesian trees for the amino acid sequence data were constructed using MrBayes v3 [25] and the parallel version of MrBayes v3 [26] under the following settings: The amino acid sequence model prior was set to mixed so that MrBayes could select the best model for the data, the number of substitution rates was set to 6, and the among site rate variation was set to be estimated under a gamma distribution. Settings for the run were the same as for the nucleotide sequence analysis. The amino acid sequence model selected by MrBayes was the Jones model [27] with a probability of 1.

### 2.3 Coiled-coiled domain identification and conservation comparison

The putative coiled-coil domain in the SIP30 protein sequence was identified with the paircoil algorithm [28]. Pair-wise amino acid sequence comparisons of SIP30 full-length sequences or coiled-coil domain sequences and the percentage of identify scores were calculated with MacVector.

## 3 Results

### 3.1 Phylogenetic analysis of mammalian SIP30 sequences

**Fig. 1:**
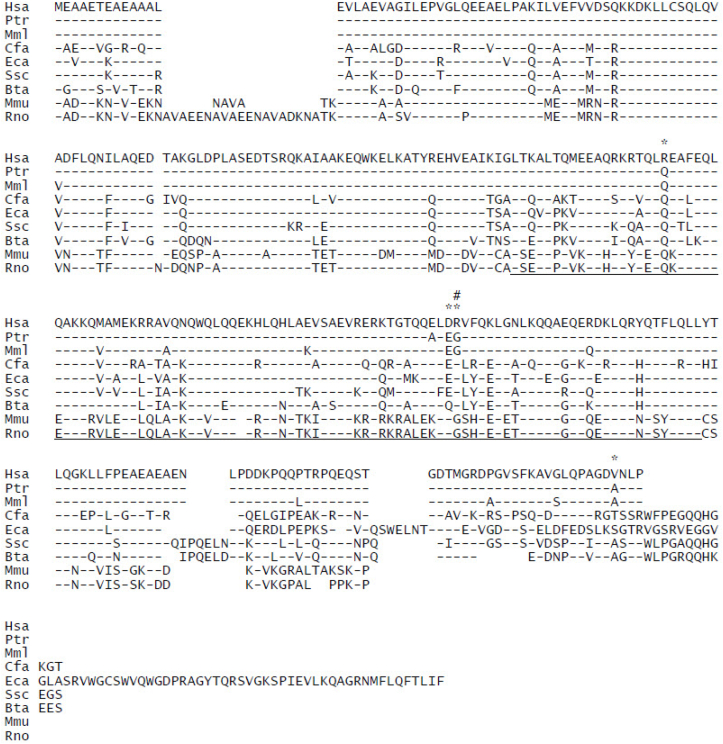
Protein sequence comparison of SIP30 orthologues. Amino acid sequences of all mammalian SIP30 available in public database are aligned. Gaps in alignment are shown as blank spaces. Amino acid residue identity with that of the corresponding human SIP30 sequence is indicated by a dash (-). * designates amino acid residues in the human SIP30 that are different from the other two primates (rhesus monkey and chimpanzee). # designates a SNP in the human SIP30 with frequency of “R” at about 30 % and “G” at about 70 %. Underline: putative coiled-coil domain. The common name, the Latin name, and the 3 letter abbreviation of each species are as following: Human—Homo sapiens—Hsa, Chimpanzee—Pan troglodytes—Ptr, Rhesus monkey—Macaca mulatta—Mml, Dog—Canis lupus familiaris—Cfa, Horse—Equus caballus—Eca, Pig—Sus scrofa—Ssc, Cow—Bos taurus—Bta, Mouse—Mus musculus—Mmu, and Rat—Rattus norvegicus-Rno.

SIP30 orthologous sequences for selected species of primates (including human), domesticated animals, and rodents were retrieved from databases and aligned at the nucleotide (data not shown) and deduced amino acid sequence levels (Fig. 1). Alignment of SIP30 protein sequences indicated that within primates, there are four human-specific amino acid residues, based on their differences from the corresponding amino acid residues in other non-human primates (Fig. 1 marked by “*“). A single nucleotide polymorphism (SNP) in the human SIP30 gene that causes a codon variation in the coiled-coil domain, resulting in either an arginine (R) or a glycine (G) at position 187 of the human SIP30 protein, at about 30 % frequency for “R” and about 70 % frequency for “G” (Fig. 1 marked by “#“). Interestingly, in aligned protein sequences, all domesticated animals have “R” at this position, while rodents and non-human primates have “G”. The functional implication of this polymorphism is unknown at present, although it is a change from a polar to a non-polar amino acid residue.

**Fig. 2:**
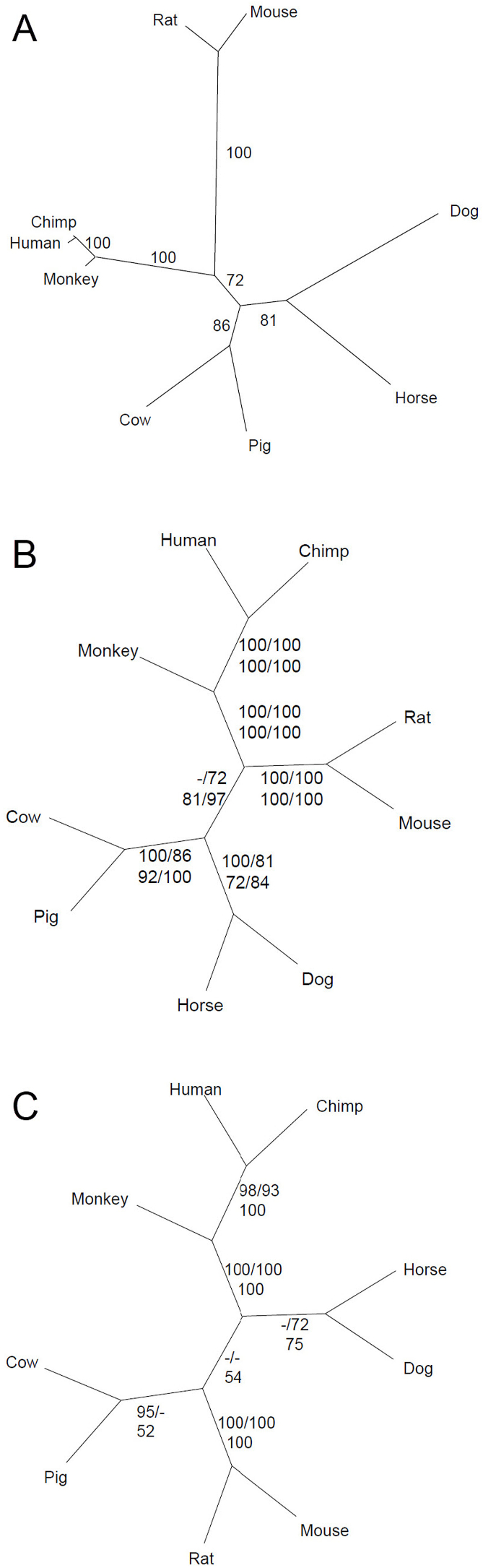
Phylogenetic relationship among SIP30 orthologues. A, The phylogenetic tree of nucleotide sequence of protein coding region was constructed using maximum parsimony method. B, Nucleotide consensus tree. The support from each tree building method is shown clockwise from top left: neighbor joining, maximum parsimony, maximum likelihood, and Bayesian. C, Protein consensus tree. The support from each tree building method is shown clockwise from top left: neighbor joining, maximum parsimony, and Bayesian.

To evaluate the evolutionary relationship among SIP30 orthologues, a number of methods were used to construct phylogenetic trees, including neighbor joining, maximum parsimony, maximum likelihood, and Bayesian posterior probability (Fig. 2). Fig. 2A shows the phylogenetic tree of nucleotide sequences for the protein coding region of SIP30, constructed using the maximum parsimony method. Notably, the human branch appears longer than that for chimpanzee, suggesting that more extensive changes happened in human during evolution, after the branching between human and chimpanzee. This phenomenon of a longer branch for human compared with chimpanzee was observed for both nucleotide and protein phylogenetic trees, for every tree-building model (data not shown). The relatively longer branch leading to humans since divergence from chimpanzee suggests a new tendency of evolutionary change in human SIP30.

Fig. 2B and C depict consensus trees derived from nucleotide and protein sequences of SIP30 orthologues, respectively, indicating the evolutionary relationships amongst these genes. Notably, SIP30 genes from rodents (mouse and rat) form a distinct group, as do SIP30 genes from primates (human, chimpanzee, and rhesus monkey), and from domesticated mammals (cow, pig, dog, and horse). Within these groups, the SIP30 genes of cow and pig appear to be more closely related than either is with dog or horse, which also form a group. Grouping based on SIP30 sequences also places rhesus monkey at the base of the primate species group. Overall, the phylogenetic analysis is consistent with the generally accepted species phylogeny, indicating an orderly evolutionary process of SIP30 genes in relation to species evolution.

### 3.2 Coiled-coil domain shows accelerated adaptation in primates

Protein sequence analysis indicated the presence of a coiled-coil domain in SIP30. Coiled-coil domains often confer the ability to interact with other proteins [29]; indeed, the coiled-coil domain of SIP30 has been reported to play an important role in its interaction with SNAP25 and Rab3C [3].

**Fig. 3:**
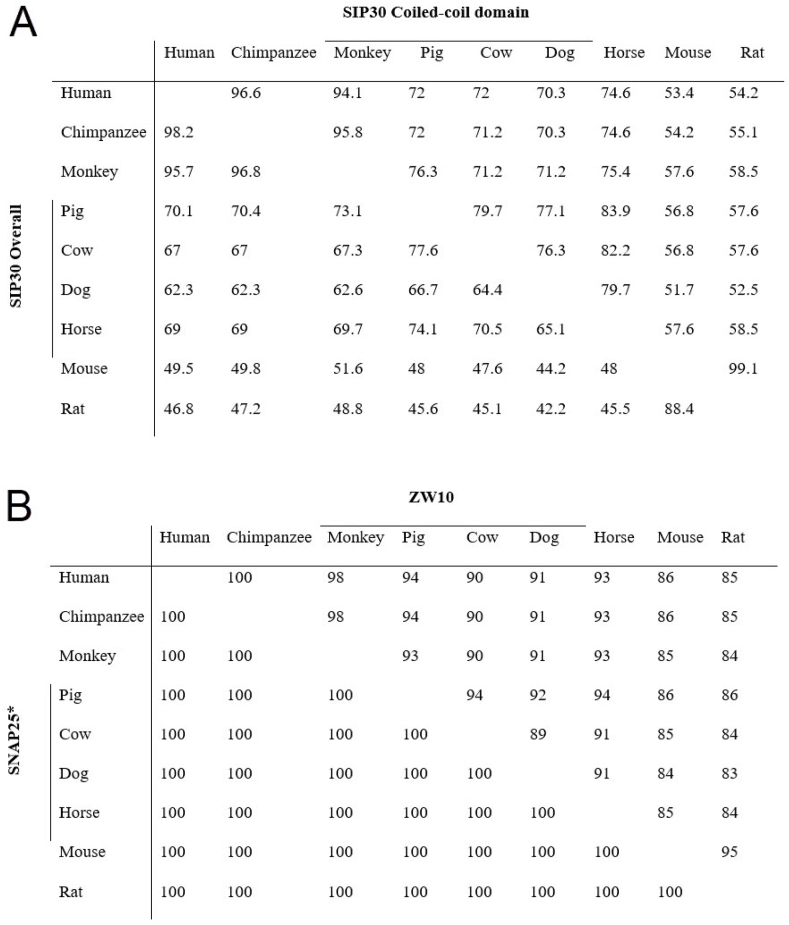
The pair-wise species comparison of amino acid residue identity as percentage values. A, For SIP30, the same species comparison values (which are always 100 %) are omitted. Comparison values for the entire SIP30 sequence are in the lower left part, and those for only the coiled-coil domain are in the top right part. B, For SNAP25 and ZW10, the same species comparison values (which are always 100 %) are omitted. Comparison values for SNAP25 sequence are in the lower left part, and those for ZW10 sequence are in the top right part. *: In some species, there are possible isoforms of SNAP25. The amino acid identity scores are still above 95 %, when the comparisons were performed with other isoforms.

Protein domains with functional importance typically exhibit higher conservation across species than the rest of the sequence. Hence, an analysis was performed on SIP30 sequences to explore this evolutionary trend. Fig. 3A shows the pair-wise species comparison of SIP30 amino acid residue identity as percentage values. The same species comparison values (which are always 100 %) are omitted, creating a diagonal blank from the top-left to the bottom-right, and dividing the values into two parts. Sequence identity values for the entire SIP30 sequence are in the lower left part, and those for only the coiled-coil domain are in the top right part.

Primates exhibit the highest level of sequence conservation among themselves, with percent identity values exceeding 90 % for both overall and coiled-coil domain sequences. They demonstrate considerably less conservation with domesticated animals, where percent identity values fall within the 60 %∼70 % range. They exhibit the lowest level of sequence conservation with rodents, showing around 50 % sequence identity values. Rodents show the highest levels of sequence conservation, displaying 99.1 % identity for the coiled-coil domain and 88.4 % identity overall. However, rodents exhibit low levels of sequence conservation with all other animals, displaying percent identity values in the 40 %∼50 % range for both primates and domesticated animals. Interestingly, the within-group high conservation tendency is not evident in domesticated animals. They display only moderate levels of sequence conservation among themselves, with percent identity values ranging from 60 % to 80 %, comparable to the values observed in the domesticated animals vs. primates.

For comparison, we conducted a sequence conservation analysis for SIP30 binding partners ZW10 [1] and SNAP25 [2], using the same 9 species in every pair-wise comparison. SNAP25 amino acid sequences exhibited 100 % identity, and ZW10 amino acid sequences showed over 80 % identity (Fig. 3B).

**Fig. 4:**
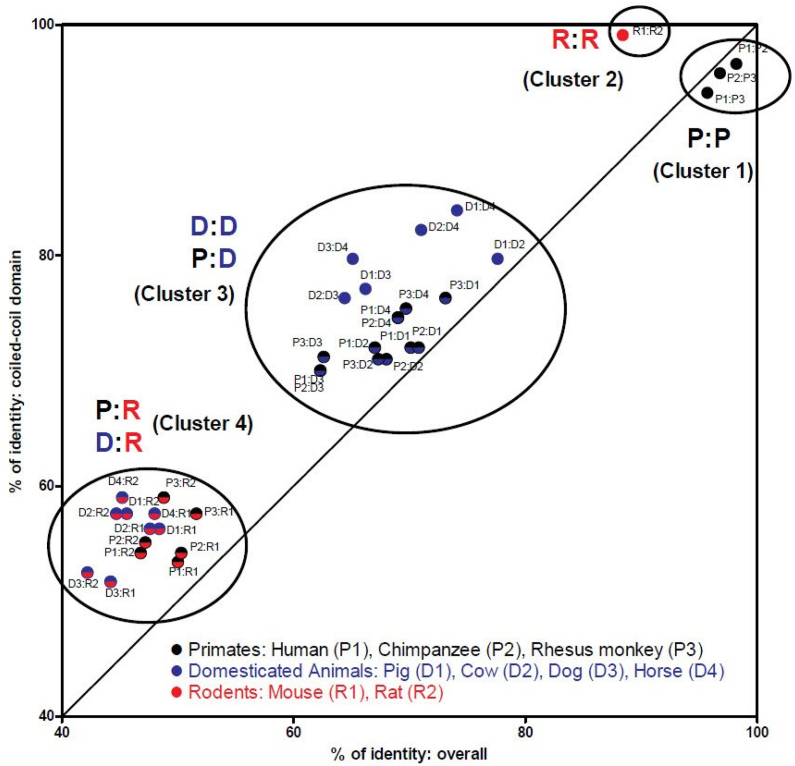
Coiled-coil domain in SIP30 gene: Evolutionary conservation among mammals vs. accelerated adaptation in primates. Data shown are geometric representation of SIP30 orthologue amino acid sequence comparisons. Percent identity values of pair-wise species comparison for the entire SIP30 protein are along the X axis, and those only for the coiled-coil domain are along the Y axis. Species are grouped and color coded: primates (human, chimpanzee, rhesus monkey) in black; domesticated animals (cow, pig, dog, horse) in blue; and rodents (mouse, rat) in red. Each data point (colored circle) represents the two comparison values between two species: overall (the whole protein) for the X axis, and coiled-coil domain only for the Y axis. Color coding highlights the species grouping by the circles. The diagonal line has a slope of 1, to visually demarcate two regions: data points above the line indicate evolutionary conservation of the coiled-coil domain over the whole protein, and those below the line indicate accelerated change in the coiled-coil domain. Data clusters are indicated by the oval shapes, which are sequentially numbered based on the mean cluster geometric value (mean distance from the 100 % identity point), with the species grouping comparison highlighted with bold-face letters. (For interpretation of the references to color in this figure legend, the reader is referred to the Web version of this article.)

Based on within-group comparisons, it is evident that primates and rodents exhibit high sequence conservation levels compared to domesticated animals, where within-group sequence conservation levels are similar to those for between-group conservation levels. This observation suggests a clustering phenomenon based on conservation value categories. To explore this clustering in greater detail, a graph was constructed for a geometric presentation of pair-wise species sequence comparisons, as illustrated in Fig. 4. For each pair-wise species comparison, the percent identity value over the entire SIP30 sequence was plotted on the X-axis, while that for the coiled-coil domain was plotted on the Y-axis.

Several features are noted. First, within-group comparisons reveal that primates (P:P) and rodents (R:R) display the highest level of sequence conservation, with the shortest distance from the complete conservation point (the top-right cross point for 100 % and 100 %), while domesticated animals (D:D) exhibit only moderate conservation. Second, in between-group comparisons, primates vs. domesticated animals show moderate conservation, similar to that observed within the domesticated animals, whereas rodents vs. either primates or domesticated animals exhibit low levels of sequence conservation. Third, a clustering effect is apparent, with D:D and P:D overlapping to form the moderate conservation cluster, and P:R and D:R overlapping to form the low conservation cluster. We computed the cluster geometric distance values from the 100 % conservation point, numbering the clusters sequentially based on these values: P:P forms cluster 1 (5.48 ± 1.23, mean ± SEM for distance values); R:R forms cluster 2 (11.63, SEM not calculated due to a single species-to-species comparison value); D:D and P:D together form cluster 3 (40.52 ± 1.26); P:R and D:R together form cluster 4 (68.89 ± 0.76).

A fourth, rather startling feature was observed with the aid of a diagonal line. This line, drawn from the complete divergence point (0 % and 0 %, not shown in Fig. 4) to the 100 % conservation point, has a slope of 1. This line represents the balanced evolution of the coiled-coil functional domain versus the entire SIP30 protein, demarcating the two regions of fundamental difference. The region above the diagonal line signifies evolutionarily conservation of the coiled-coil domain over the entire protein and includes all pair-wise species comparisons in Clusters 2, 3 and 4. Conversely, the region below the diagonal line indicates more extensive divergence of the coiled-coil domain than the entire protein sequence -- meaning mutations accumulate at a faster rate within the coiled-coil functional domain than for the rest of the protein. Intriguingly, this region encompasses all P:P comparisons (Cluster 1), suggesting accelerated adaptation in the coiled-coil functional domain among primates.

## 4 Discussion

In this study, we examined the SIP30 gene within selected mammalian species among three distinct groups (primates, domesticated animals, and rodents). We investigated its orthologue sequence divergence among these species, analyzed its phylogenetic tree structure, and explored the relative evolution rate of its functional domain compared to the entire protein.

SIP30 was initially identified for its interaction with ZW10 and was consequently named Zwint (ZW10 interacting protein) [1], a kinetochore protein involved in the mitotic spindle checkpoint. Subsequent studies revealed interactions between SIP30 and two other proteins, SNAP25 and Rab3 [2,3], which play critical and regulatory roles in calcium-dependent synaptic vesicle exocytosis, suggesting potential involvement of SIP30 in this process. We showed that when the expression of SIP30 in PC12 cells was inhibited by siRNA transfection, the stimulated exocytosis was inhibited [8,14], and that SIP30 gene was regulated by ERK during neuropathic pain [9].

In the current study, we observed a substantial degree of sequence divergence in SIP30 when compared to its binding partners ZW10 and SNAP25, which exhibit remarkable conservation across species with minimal sequence divergence [30,31]. In the nine species examined in this study, every pair-wise comparison of SNAP25 amino acid sequences showed 100 % identity, while ZW10 amino acid sequences showed over 80 % identity (Fig. 3B). In contrast, SIP30 exhibited amino acid sequence identity values as low as 40–50 % (Fig. 3A).

Within primates, the coiled-coil functional domain in the SIP30 protein exhibits greater sequence divergence compared to the entire protein. In evolution terms, functional protein motifs, such as the coiled-coil domain, are typically more conserved than the rest of the protein, reflecting the selective pressure to maintain the functional domain. This pattern is evident in SIP30 orthologue pair-wise comparisons among all non-primates and in primates vs. non-primates (Fig. 4, Clusters 2, 3, and 4, above the diagonal line denoting balanced evolution), regardless of whether the pair-wise comparison indicates high conservation (Cluster 2), moderate conservation (Cluster 3), or low conservation (Cluster 4).

However, pair-wise comparisons of SIP30 orthologues among primates display more divergence for the coiled-coil domain than the rest of the protein (Fig. 4, Cluster 1, below the diagonal line). This suggests that among primates, the coiled-coil functional domain accumulated mutations at a faster rate than the rest of the protein, indicating accelerated adaptation of this functional domain in primates.

SIP30 is involved in sensory synaptic processing and plays an important role in neuropathic pain [[8], [9], [10]]. Neuropathic pain, affecting 7 %–15 % of the general population [[32], [33], [34], [35]], poses a considerable clinical challenge with unsatisfactory treatment options [12,15,36,37]. Despite persistent effort to develop drugs for neuropathic pain, the field has encountered a high failure rate [38].

Species differences in the nervous system contribute to the challenges in translating discovery research into effective therapies for neuropathic pain, given that preclinical studies in rodents and other non-primate models may not accurately predict therapeutic efficacy in humans [38]. Since abnormal firing of sensory neurons is a fundamental cause of neuropathic pain [11], and SIP30 modulates synaptic vesicle exocytosis [14], a key process in synaptic activity, investigating the molecular components of the synaptic machinery underlying species differences is crucial.

This report demonstrates that SIP30 exhibits significant sequence divergence across mammalian species, with accelerated adaptation of functional domain in primates. This is in contrast to the low degrees of sequence divergence observed in SNAP25 and ZW10, two important molecules in exocytosis and binding partners of SIP30. This suggests that SIP30 may be a part of a molecular mechanism contributing to species specificity in synaptic function. Consequently, further investigation of SIP30 (and other highly divergent synaptic molecules) as potential targets is warranted, offering the prospect of providing novel and effective therapies for alleviating neuropathic pain and related suffering in affected human patients.

## Funding

This work was supported in part by grants from the 10.13039/100000002National Institutes of Health of the United States (DA013471 and DA020555 to L.Y.), and 10.13039/100005200New Jersey Commission on Spinal Cord Research (10-3093-SCR-E-0 to L.Y.).

## CRediT authorship contribution statement

**Ning Guo:** Writing – original draft, Methodology, Investigation, Formal analysis, Data curation, Conceptualization. **Jeremy Raincrow:** Writing – original draft, Software, Methodology, Investigation, Formal analysis, Data curation. **Chi-hua Chiu:** Writing – review & editing, Writing – original draft, Supervision, Project administration. **Lei Yu:** Writing – review & editing, Writing – original draft, Supervision, Project administration, Funding acquisition, Conceptualization.

## Declaration of competing interest

The authors declare no conflict of interest.

## References

[1] Starr D.A., Saffery R., Li Z., Simpson A.E., Choo K.H., Yen T.J., Goldberg M.L. (2000). HZwint-1, a novel human kinetochore component that interacts with HZW10. J. Cell Sci..

[2] Lee H.K., Safieddine S., Petralia R.S., Wenthold R.J. (2002). Identification of a novel SNAP25 interacting protein (SIP30). J. Neurochem..

[3] van Vlijmen T., Vleugel M., Evers M., Mohammed S., Wulf P.S., Heck A.J., Hoogenraad C.C., van der S.P. (2008). A unique residue in rab3c determines the interaction with novel binding protein Zwint-1. FEBS Lett..

[4] Südhof T.C., Rothman J.E. (2009). Membrane fusion: grappling with SNARE and SM proteins. Science.

[5] Jahn R., Cafiso D.C., Tamm L.K. (2023). Mechanisms of SNARE proteins in membrane fusion. Nat. Rev. Mol. Cell Biol..

[6] Fischer v.M., Stahl B., Li C., Sudhof T.C., Jahn R. (1994). Rab proteins in regulated exocytosis. Trends Biochem. Sci..

[7] Schluter O.M., Khvotchev M., Jahn R., Sudhof T.C. (2002). Localization versus function of Rab3 proteins. Evidence for a common regulatory role in controlling fusion. J. Biol. Chem..

[8] Zhang Y., Guo N., Peng G., Wang X., Han M., Raincrow J., Chiu C., Coolen L.M., Wenthold R.J., Zhao Z., Jing N., Yu L. (2009). Role of SIP30 in the development and maintenance of peripheral nerve injury-induced neuropathic pain. Pain.

[9] Peng G., Han M., Du Y., Lin A., Yu L., Zhang Y., Jing N. (2009). SIP30 is regulated by ERK in peripheral nerve injury-induced neuropathic pain. J. Biol. Chem..

[10] Han M., Xiao X., Yang Y., Huang R.Y., Cao H., Zhao Z.Q., Zhang Y.Q. (2014). SIP30 is required for neuropathic pain-evoked aversion in rats. J. Neurosci..

[11] Xie W., Strong J.A., Meij J.T., Zhang J.M., Yu L. (2005). Neuropathic pain: early spontaneous afferent activity is the trigger. Pain.

[12] Dworkin R.H., O'Connor A.B., Kent J., Mackey S.C., Raja S.N., Stacey B.R., Levy R.M., Backonja M., Baron R., Harke H., Loeser J.D., Treede R.D., Turk D.C., Wells C.D. (2013). Interventional management of neuropathic pain: NeuPSIG recommendations. Pain.

[13] Scholz J., Finnerup N.B., Attal N., Aziz Q., Baron R., Bennett M.I., Benoliel R., Cohen M., Cruccu G., Davis K.D., Evers S., First M., Giamberardino M.A., Hansson P., Kaasa S., Korwisi B., Kosek E., Lavand'homme P., Nicholas M., Nurmikko T., Perrot S., Raja S.N., Rice A.S.C., Rowbotham M.C., Schug S., Simpson D.M., Smith B.H., Svensson P., Vlaeyen J.W.S., Wang S.J., Barke A., Rief W., Treede R.D. (2019). The IASP classification of chronic pain for ICD-11: chronic neuropathic pain. Pain.

[14] Guo N., Yu L. (2023). SIP30 involvement in vesicle exocytosis from PC12 cells. Biochemistry and Biophysics Reports.

[15] Kankowski S., Grothe C., Haastert-Talini K. (2021). Neuropathic pain: spotlighting anatomy, experimental models, mechanisms, and therapeutic aspects. Eur. J. Neurosci..

[16] Bouali-Benazzouz R., Landry M., Benazzouz A., Fossat P. (2021). Neuropathic pain modeling: focus on synaptic and ion channel mechanisms. Prog. Neurobiol..

[17] White D., Abdulla M., Park S.B., Goldstein D., Moalem-Taylor G., Lees J.G. (2023). Targeting translation: a review of preclinical animal models in the development of treatments for chemotherapy-induced peripheral neuropathy. J. Peripher. Nerv. Syst..

[18] Posada D., Crandall K.A. (2007). MODELTEST: testing the model of DNA substitution. Bioinformatics.

[19] Raftery A.E. (1986). Choosing models for cross-classification. Am. Socio. Rev..

[20] Raftery A.E. (1986). A note on Bayes factors for log-linear contingency table models with vague prior information. J Royal Stat Soc Series B.

[21] Hasegawa M., Kishino H., Yano T. (1985). Dating of the human-ape splitting by a molecular clock of mitochondrial DNA. J. Mol. Biol..

[22] Nylander J.A.A. (2004).

[23] Swofford D.L. (2003).

[24] Zwickl D.J. (2006).

[25] Ronquist F., Huelsenbeck J.P. (2003). MrBayes 3: Bayesian phylogenetic inference under mixed models. Bioinformatics.

[26] Altekar G., Dwarkadas S., Huelsenbeck J.P., Ronquist F. (2004). Parallel Metropolis coupled Markov chain Monte Carlo for Bayesian phylogenetic inference. Bioinformatics.

[27] Jones D.T., Taylor W.R., Thornton J.M. (1992). The rapid generation of mutation data matrices from protein sequences. Comput. Appl. Biosci..

[28] Berger B., Wilson D.B., Wolf E., Tonchev T., Milla M., Kim P.S. (1995). Predicting coiled coils by use of pairwise residue correlations. Proc. Natl. Acad. Sci. U.S.A..

[29] Lupas A. (1996). Coiled coils: new structures and new functions. Trends Biochem. Sci..

[30] Risinger C., Blomqvist A.G., Lundell I., Lambertsson A., Nassel D., Pieribone V.A., Brodin L., Larhammar D. (1993). Evolutionary conservation of synaptosome-associated protein 25 kDa (SNAP-25) shown by Drosophila and Torpedo cDNA clones. J. Biol. Chem..

[31] Starr D.A., Williams B.C., Li Z., Etemad-Moghadam B., Dawe R.K., Goldberg M.L. (1997). Conservation of the centromere/kinetochore protein ZW10. J. Cell Biol..

[32] van Hecke O., Austin S.K., Khan R.A., Smith B.H., Torrance N. (2014). Neuropathic pain in the general population: a systematic review of epidemiological studies. Pain.

[33] Colloca L., Ludman T., Bouhassira D., Baron R., Dickenson A.H., Yarnitsky D., Freeman R., Truini A., Attal N., Finnerup N.B., Eccleston C., Kalso E., Bennett D.L., Dworkin R.H., Raja S.N. (2017). Neuropathic pain. Nat. Rev. Dis. Prim..

[34] Cavalli E., Mammana S., Nicoletti F., Bramanti P., Mazzon E. (2019). The neuropathic pain: an overview of the current treatment and future therapeutic approaches. Int. J. Immunopathol. Pharmacol..

[35] Soliman N., Kersebaum D., Lawn T., Sachau J., Sendel M., Vollert J. (2023). Improving neuropathic pain treatment - by rigorous stratification from bench to bedside. J. Neurochem..

[36] Dworkin R.H., O'Connor A.B., Backonja M., Farrar J.T., Finnerup N.B., Jensen T.S., Kalso E.A., Loeser J.D., Miaskowski C., Nurmikko T.J., Portenoy R.K., Rice A.S., Stacey B.R., Treede R.D., Turk D.C., Wallace M.S. (2007). Pharmacologic management of neuropathic pain: evidence-based recommendations. Pain.

[37] O'Connor A.B., Dworkin R.H. (2009). Treatment of neuropathic pain: an overview of recent guidelines. Am. J. Med..

[38] Baron R., Dickenson A.H., Calvo M., Dib-Hajj S.D., Bennett D.L. (2023). Maximizing treatment efficacy through patient stratification in neuropathic pain trials. Nat. Rev. Neurol..

