# Supplementary material for: Phylogenetic analysis of mammalian SIP30 sequences indicating accelerated adaptation of functional domain in primates
Source: Biochem Biophys Rep. 2024 Jan 1;37:101631. doi: 10.1016/j.bbrep.2023.101631 (PMC10771893; doi:10.1016/j.bbrep.2023.101631)
Supplement: Multimedia component 1 [file mmc1.docx]

**Supplemental Tables**

Supplemental Table 1. SIP30 nucleotide and amino acid sequences from the mammalian species used in this study.

| Species | ACCESSION# | Protein ID |
| --- | --- | --- |
| Homo sapiens (human) | NM_032997 | NP_127490.1 |
| Pan troglodytes (chimpanzee) | XM_507799 | XP_507799.2 |
| Macaca mulatta (rhesus monkey) | XM_001098136 | XP_001098136.1 |
| Sus scrofa (pig) | AK236975 | XP_013838948.1 |
| Bos taurus (cow) | NM_001040529 | NP_001035619.1 |
| Equus caballus (horse) | XM_001501939 | XP_001501989 |
| Canis familiaris (dog) | XM_534774 | XP_534774.2 |
| Mus musculus (mouse) | NM_025635 | NP_079911.1 |
| Rattus norvegicus (rat) | NM_147138 | NP_671479.1 |

Supplemental Table 2. List of SNAP25 and ZW10 amino acid sequences from different species used in sequence comparison.

|  | SNAP25 | ZW10 |
| --- | --- | --- |
| Homo sapiens (human) | NP_570824.1 | NP_004715.1 |
| Pan troglodytes (chimpanzee) | NP_001009094.1 | XP_508761.2 |
| Macaca mulatta (rhesus monkey) | NP_001028036.1 | XP_001085694.1 |
| Sus scrofa (pig) | XP_020934018.1 | XP_020918499.1 |
| Bos taurus (cow) | NP_001069714.1 | NP_001068957.1 |
| Equus caballus (horse) | XP_001493159.1 | XP_001502103.1 |
| Canis familiaris (dog) | XP_534347.1 | XP_536569.2 |
| Mus musculus (mouse) | NP_035558.1 | NP_036169.1 |
| Rattus norvegicus (rat) | NP_112253.1 | NP_001019972.1 |
